# Theoretical and Experimental Vibrational Characterization of Biologically Active Nd(III) Complex

**DOI:** 10.3390/molecules26092726

**Published:** 2021-05-06

**Authors:** Irena Kostova, Jan Mojžiš, Vasile Chiş

**Affiliations:** 1 Department of Chemistry, Faculty of Pharmacy, Medical University, 2 Dunav St., 1000 Sofia, Bulgaria; i.kostova@pharmfac.mu-sofia.bg; 2 Department of Pharmacology, Faculty of Medicine, P.J. Šafarik University, 04011 Košice, Slovakia; jan.mojzis@upjs.sk; 3 Department of Biomolecular Physics, Faculty of Physics, Babeş-Bolyai University, 400084 Cluj-Napoca, Romania

**Keywords:** Nd(III) complex, orotic acid, IR, Raman, DFT, cytotoxicity

## Abstract

The neodymium(III) complex of orotic acid (HOA) was synthesized and its structure determined by means of analytical and spectral analyses. Detailed vibrational analysis of HOA, sodium salt of HOA, and Nd(III)–OA systems based on both the calculated and experimental spectra confirmed the suggested metal–ligand binding mode. Significant differences in the IR and Raman spectra of the complex were observed as compared to the spectra of the ligand. The calculated vibrational wavenumbers, including IR intensities and Raman scattering activities, for the ligand and its Nd(III) complex were in good agreement with the experimental data. The vibrational analysis performed for the studied species, orotic acid, sodium salt of orotic acid, and its Nd(III) complex helped to explain the vibrational behaviour of the ligand’s vibrational modes, sensitive to interaction with Nd(III). In this paper we also report preliminary results about the cytotoxicity of the investigated compounds. The cytotoxic effects of the ligand and its Nd(III) complex were determined using the MTT method on different tumour cell lines. The screening performed revealed that the tested compounds exerted cytotoxic activity upon the evaluated cell lines.

## 1. Introduction

Lanthanide complexes are of great interest because of their various potential applications. Due to the unique nature of lanthanide ions, such as their large radius and high coordination number, the assembly of lanthanide complexes possessing novel structures and special properties offers great challenges and opportunities in terms of controlling their shapes and dimensions. The selection of an appropriate organic ligand along with different synthetic methods is a key step in the construction of lanthanide complexes with the desired features. Ligands containing a combination of nitrogen and oxygen donor atoms demonstrate flexible coordination modes during the formation of coordination frameworks, which is why the studied lanthanide(III) complexes of biologically active derivatives of orotic acid deserve to be examined.

**Figure 1:**
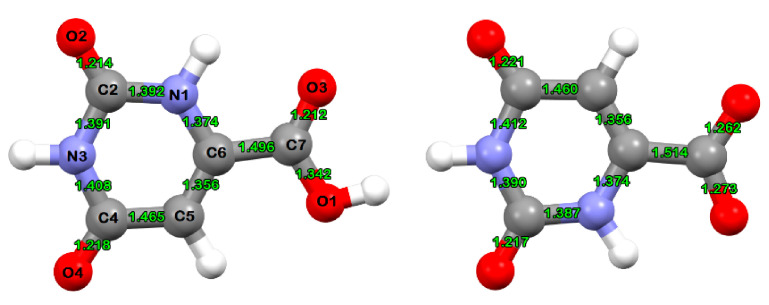
B3LYP/cc-pVDZ(SDD)-optimized molecular structures of the orotic acid ligand (**left**) and the orotic acid anion (**right**).

The coordination chemistry of orotic acid (2,6-dioxo-1,2,3,6-tetrahydropyrimidine-4-carboxylic acid, vitamin B_13_, or HOA) and its sodium salt (NaOA) (see Figure 1) has been an area of great interest [1,2,3,4,5], ranging from bioinorganic to pharmaceutical and materials chemistry.

Metal orotates are widely applied in medicine [6], with platinum, palladium, and nickel orotate complexes being screened as potential therapeutic agents for cancer [7]. More recent interest has focused on the proposed biological function of orotic acid and its corresponding anions in binding biogenic metal ions, which is held responsible for the successful application of orotate complexes in curing syndromes associated with a deficiency of a variety of metals—such as Ca, Mg, Zn, or Fe—thus delivering these metals conveniently to patients [4,5,6]. However, previous studies on the coordination chemistry of orotic acid mainly focused on transition metals or alkali metals, while the lanthanides have been neglected.

Aside from the significance of orotic acid and its derivatives in biological systems, these compounds possess fascinating coordination behaviour with potential hydrogen-bonding interactions, such as asymmetric geometry and multiple coordination sites. Orotic acid acts as a diacid in aqueous solution [8,9]. The multifunctionality of its anions offers interesting possibilities in crystal engineering as a versatile ligand for supramolecular assemblies. The coordinated orotate anions exhibit a ligand surface with double or triple hydrogen-bonding capabilities, depending on the metal coordination mode, and thus have the potential to adopt several modes of interligand hydrogen bonding to allow for the formation of extended, self-assembled structures. Orotic acid has demonstrated versatile coordination modes during the formation of coordination frameworks, which is why it was a challenge for us to obtain new lanthanide(III) coordination complexes with this ligand, especially in view of their application as anticancer agents. To the best of our knowledge, little is known about lanthanide(III) coordination compounds with orotic acid, and such complexes possessing cytotoxic activity has not been previously reported. We have recently synthesized lanthanide complexes with a number of biologically active ligands, and we reported their significantly high cytotoxic activity in different human tumour cell lines [10,11,12,13,14,15,16,17,18]. These promising results prompted us to search for new lanthanide complexes with orotic acid. Thus, the aim of this work was to synthesize and characterize a complex of neodymium(III) with orotic acid and, subsequently, to evaluate its cytotoxic activity.

The present work is a continuation of our earlier long experience with lanthanide complexes. In this paper we report a joint theoretical, analytical, and spectroscopic study on the new Nd(III) complex of orotic acid (HOA). The Nd(III)–OA binding mode was identified and characterized via elemental analysis, along with both IR and Raman spectroscopy, coupled with quantum chemical calculations based on density functional theory (DFT). The cytotoxic effects of the ligand and its Nd(III) complex were determined via the MTT method using different tumour cell lines. Recently we have reported thorough studies on the optimized geometries and vibrational characterization of different Ln(III) model systems (some of them similar to the investigated one) to suggest the metal–ligand binding mode by using high-level theoretical methods [10,11,12,13,14,15,16,17,18,19]. This detailed theoretical approach helped to predict correctly the metal coordination polyhedron of the studied complex. The recorded spectra of the Ln(III) complexes are very similar, and this finding is indicative of similar binding of the ligand and similar coordination polyhedrons of the complexes. The applied methodology proved to be reliable for the series of lanthanide complexes, and the results are in good agreement with those existing in the literature. Since the crystal structure data are not available, theoretical approaches for determination of the geometrical parameters, vibrational frequencies, hydrogen bond strengths, and the binding mode for the model Ln(III)–OA at a high level of theory are very helpful for extracting reliable structural information.

## 2. Results and Discussion

### 2.1. Chemistry

The new complex was characterized via elemental analysis. The content of the metal ion was determined after mineralization. The water content of the complex was determined via Karl Fischer titration and thermogravimetric analysis. IR and Raman spectra confirmed the nature of the complex.

The obtained elemental analysis data of the new neodymium(III) complex served as the basis for the determination of its empirical formula, and the results of the Karl Fischer titration are presented below.

Elemental analysis of the Nd(III) complex of orotic acid: (% calculated/found): Nd(OA)_2_(OH).3H_2_O: C: 22.86/23.05; H: 2.48/2.69; N: 10.66/10.37; H_2_O: 10.28/9.88; Nd: 27.43/27.15, where HOA = C_5_N_2_O_4_H_4_ and OA^−^ = C_5_N_2_O_4_H_3_^−^.

The mode of bonding of the ligand to the Nd(III) ions was elucidated by recording the IR and Raman spectra of the complex and comparing them with those of the free ligand and with the theoretical predictions. The vibrational band assignments for the ligand [19] and the Nd(III) complex were made on the basis of DFT calculations by comparison with the results reported previously in the literature [20,21,22,23,24,25,26].

### 2.2. Geometry Optimization

Because no crystal structure data were available for the Nd(III) complex of orotic acid, its structure was optimized at the B3LYP/cc-pVDZ(SDD) level of theory and compared with the literature data for compounds containing identical or similar functional groups [27].

The determination of the binding mode on the basis of physicochemical and spectroscopic methods, when crystal and molecular structure data are not available, is not a trivial task. Unfortunately, due to poor solubility we did not succeed in obtaining a single crystal suitable for X-ray diffraction analysis and, hence, the complex crystal and molecular structures are not known. Therefore, we undertook a combined theoretical and experimental study aiming to determine the binding mode of the ligand and the molecular geometry of its complex.

Different binding modes have been tested for the Nd(III) complex, and the one reported here reproduced the best available experimental vibrational data. For all other presumed binding modes it was either not possible to achieve the geometric convergence (NdIII)–2OA–2H_2_O, neutral, singlet) or else the calculated vibrational spectra predicted one or more imaginary frequencies (Nd(III)–2OA–H_2_O–OH complex, neutral, doublet) or provided very bad agreement with the experimental data (Nd(III)–2OA–H_2_O–OH complex, mono-cation, singlet).

Moreover, the calculated vibrational spectra for the proposed complex are in line with the data reported for a similar binding mode of a Co(II)-orotate complex reported by Brockner et al. (see Table 4 in Reference [28]).

The calculated Nd–O distances of the new Nd(III) complex of orotic acid are listed in Table 1. The Nd(III) ion is at least three-coordinate, with bonds to the oxygen atoms from the carboxylates of the orotate ligands and to the oxygen atom from the hydroxyl anion. The complex is mononuclear, and each local coordination unit around the metal ion contains two orotate ligands and one OH^−^ ion. The calculated partial atomic polar tensor (APT) charges on the Nd and O atoms are also included in Table 1.

### 2.3. Vibrational Spectroscopy

In Table 2 are reported selected calculated and experimental IR and Raman data, together with their tentative assignments. The last column in Table 1 contains the motions that contribute the most to different normal modes of the Nd(III) complex, according to B3LYP/cc-pVDZ(SDD) theoretical data.

The computed wavenumbers have been scaled by 0.971 [29]. To aid in mode assignment, we based this on the direct comparison between the experimental and calculated spectra, by considering both the frequency sequence and the intensity pattern.

**Figure 2:**
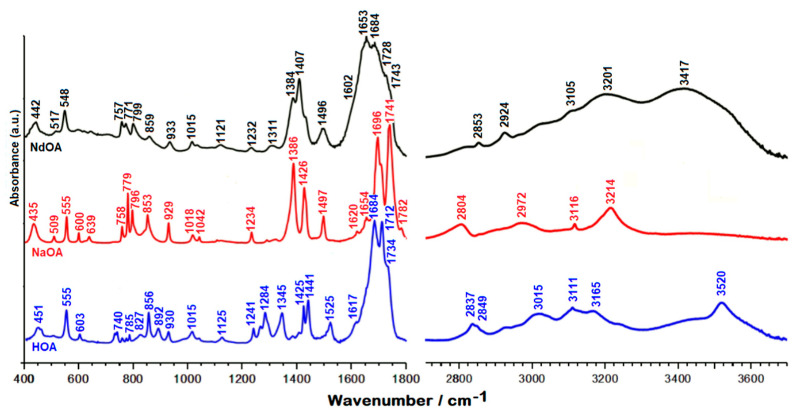
IR spectra of orotic acid (HOA), sodium salt of orotic acid (NaOA), and its Nd(III) complex (NdOA).

**Figure 3:**
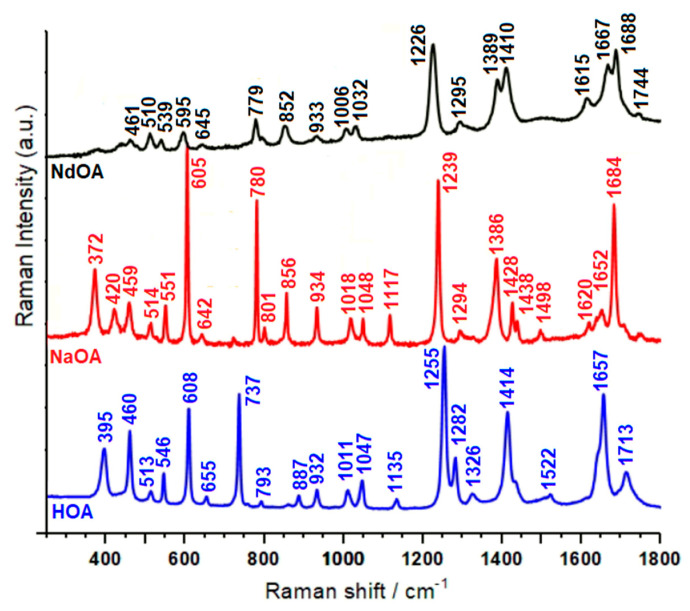
Raman spectra of the solid state of orotic acid (HOA), sodium salt of orotic acid (NaOA), and its Nd(III) complex. Excitation: 784.8 nm, 35 mW.

The vibrational IR and Raman spectra of HOA, sodium salt of orotic acid (NaOA), and Nd(III)–OA are presented in Figure 2 and Figure 3.

In the 3600–2000 cm^−1^ spectral region from the IR spectrum, the O–H and N–H stretches give rise to medium IR bands (Figure 2). The assignment of the O–H and N–H bands is quite difficult because they appear to overlap in the same spectral region, and the involvement of these groups in hydrogen bonds affects their wavenumbers and produces a relevant band broadening [21,23,24,25,26]. In the IR spectra, the medium bands at 3520 cm^−1^ (orotic acid) and 3417 cm^−1^ (Nd(III) complex of orotic acid) were assigned to the N–H stretching modes, while the shoulder at 3232 cm^−1^ (orotic acid) and the medium band at 3201 cm^−1^ (Nd(III) complex of orotic acid) were attributed to the O–H stretching modes (Table 2).

When the carbonyl group is hydrogen bonded but not dimerized, active IR and Raman bands are expected at 1730–1705 cm^−1^. In our IR spectra (Figure 2), one very strong band can be observed in this region (at 1712 cm^−1^ for orotic acid), which was assigned to the C=O stretching modes. In the same region we observed one medium band at 1713 cm^−1^ in the Raman spectrum of the free ligand, and two shoulders at 1743 and 1728 cm^−1^ in the Raman spectrum of the complex (Figure 3) [27,30,31,32,33]. According to theoretical data shown in Table 2, these vibrations can safely be assigned to the C=O stretching modes of carbonyl groups.

As shown in Figure 2 and Figure 3, in the 1600–1700 cm^−1^ region, both the IR and Raman spectra of the complex are changed drastically with respect to the free orotic acid. This change is to be expected assuming the coordination of the HOA to the Nd(III) ion through the COO^−^ group of HOA. The very strong bands observed at 1684 and 1688 cm^−1^ in the IR and Raman spectra of the Nd(III) complex were assigned to the asymmetric C=O stretching mode. In the case of unidentate ligands, the symmetric stretch of the COO group is expected to give a band in the 1420–1260 cm^−1^ region [34]. Indeed, for the Nd(III) complex we observe this vibration at 1407 and 1411 cm^−1^ in the IR and Raman spectra, respectively. Computational data support this assignment, with the vibration being predicted at 1399 cm^−1^, coupled to the C–C stretching modes. These findings are in agreement with the data reported by Dobblaere et al. for glycolato(-peroxo)–Ti(IV) complexes [35]. Moreover, the difference between the wavenumbers corresponding to the asymmetric and symmetric COO stretching modes is 277 cm^−1^, in perfect agreement with previously reported data for complexes with one monodentate and one bidentate ligand.

Another band indicative of monodentate and bidentate coordination of Nd(III) ions is the very strong band corresponding to the C7–O1 stretch, which is observed in the Raman spectrum at 1226 cm^−1^ and as a weak band at 1232 cm^−1^ in the IR spectrum. As seen in Table 2, this is significantly red-shifted with respect to the case of free OAH [19].

The C=C double bond stretching modes in the two heterocycles give rise to medium-strong absorptions observed for the Nd(III) complex in the Raman spectrum at 1667 and 1615 cm^−1^, and in the IR spectrum at 1653 and 1602 cm^−1^. The positions of these bands remain very close to those observed for the free ligand.

A characteristic band for the complex but absent for the free ligand is that seen in the IR spectrum at 1496 cm^−1^, also observed as a very weak and broad band in the Raman spectrum at 1502 cm^−1^. According to quantum chemical calculations this is mainly due to the N1H bending, coupled to C6N1 stretching.

The C5–H5, N1–H1, and C–O–H bending modes are present in the IR spectrum at 1284 cm^−1^ for the free ligand, while in the Raman spectrum they are detected at 1282 cm^−1^ and 1295 cm^−1^, respectively. The weak band at 1266 cm^−1^, which can be observed only in the IR spectrum of the orotic acid, can be due to the C=O bending mode and to the symmetrical stretching mode of N1–C2–N3. According to theoretical data, the bands around 1015 cm^−1^, weak in IR and medium in Raman spectra, are due to in-plane deformations of uracilate rings, whereas bands around 930 cm^−1^, almost weak in IR and medium in Raman spectra, were attributed to the symmetrical C(ring)–C(carboxyl) bridge bond stretching mode.

The uracilate ring bending vibration and the skeletal deformation bands of the free orotic acid, mainly in the 900–300 cm^−1^ wavenumber region, show considerable changes in complex formation. These changes may be attributed to distortion of the uracilate rings upon coordination.

The medium band at 779 cm^−1^ seen in the Raman spectrum of the complex is due to the bending of the carboxylate group coupled with the Nd–O stretching. Moreover, the Nd–O stretching contributes greatly to the medium band observed in the Raman spectrum of the complex at 595 cm^−1^.

The new bands at 442 cm^−1^ in the IR spectrum and at 440 cm^−1^ (very weak) in the Raman spectrum, which appear only for the Nd(III) complex, are due to the neodymium–oxygen stretching [21,22,26], being predicted at 459 cm^−1^ by DFT calculations. In the low wavenumbers region of the Raman spectrum of orotic acid (Figure 3), the medium strong band at 395 cm^−1^ is shifted to the shorter wavenumbers in the Raman spectrum of the Nd(III) complex (373 cm^−1^), and becomes weaker. As seen in Table 2, this band is due to δ(OCN) deformations coupled with Nd–O stretching, according to the literature data [36,37,38,39,40]. The metal affects the carboxylate anion as well as the ring structure. The ionic potential [41] of the metal is the most important parameter responsible for the influence of the metal on the rest of the molecule [42,43,44]. The carboxylic acids interact with the metals as symmetric [45,46] bidentate carboxylate anions, and both oxygen atoms of the carboxylate are symmetrically bonded to the metal [47]. In this sense, we can observe in the Raman spectrum of the Nd(III) complex a medium weak peak at 211 cm^−1^, which, according to the computational data, is due to the O–Nd–O vibration modes (Table 2) [40,48,49,50].

In conclusion, the complex described above demonstrates once more the versatility of the orotate ligand, which adopts different coordination modes. The different charge and coordination modes of the ligand have a major effect on the supramolecular structures adopted by the complex. From previous results and this work, it is clear that the nature of orotic acid makes its various anionic forms versatile ligands for use with a variety of metals and for a variety of objectives/advantages, including variable coordination modes, high-nuclearity aggregate formation, and/or linking of aggregates into polymeric arrays. Thus, orotic acid has great potential as a generally useful new polyfunctional ligand in metal chemistry, and will prove attractive to a variety of coordination chemists.

**Figure 4:**
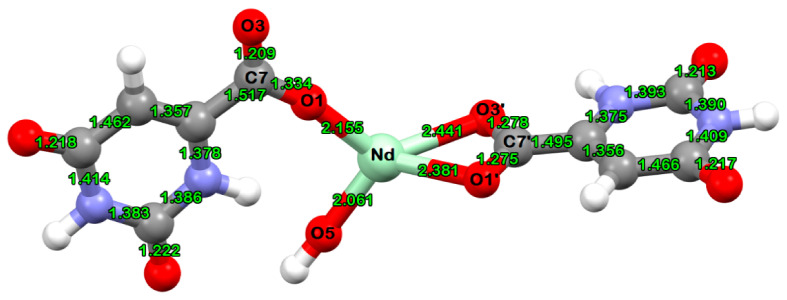
B3LYP/cc-pVDZ(SDD)-optimized molecular structure of the Nd(III) complex of orotic acid.

On the basis of the detailed vibrational analysis, the most probable structure of the obtained Nd(III) complex was predicted, and is shown in Figure 4.

### 2.4. Pharmacology

The results of the preliminary cytotoxic screening of orotic acid, sodium salt of orotic acid, and the Nd(III) complex are presented in Table 3. The investigated compounds were tested for cytotoxic activity on the Jurkat (acute T-lymphoblastic leukemia, bcl-2 overexpressed), CCRF-CEM (acute T-lymphoblastic leukemia), HeLa (human cervical adenocarcinoma), A-549 (lung carcinoma), MCF-7 (mammary gland adenocarcinoma, oestrogen receptor expressed), and MDA-MB-231 (human breast adenocarcinoma, oestrogen receptor-negative) cell lines. The results obtained indicate that the tested compounds exerted cytotoxic activity upon the evaluated cell lines.

These results confirmed our previous observations on the antioxidant activity and cytotoxicity of lanthanide(III) complexes with other biologically active ligands, such as 5-aminoorotic acid [51,52,53]. In the literature little was known about Ln(III) coordination compounds with orotic and 5-aminoorotic acid, and such complexes possessing antioxidant and anticancer activity had not been previously reported. The present work can be regarded as a continuation of our efforts in the bioinorganic chemistry of lanthanide(III) complexes with a number of biologically active ligands.

## 3. Materials and Methods

### 3.1. Synthesis of the Coordination Complex

The compounds used for preparing the solutions were Merck products, pro analysis grade, Nd(NO_3_)_3_.6H_2_O. The sodium salt of orotic acid was used for the preparation of the metal complex as a ligand.

The complex was synthesized via the reaction of neodymium(III) nitrate and the sodium salt of orotic acid in aqueous solution, in amounts equal to a metal/ligand molar ratio of 1:2. The formation of the complex may be represented by the following equations:

NaOA + H_2_O ↔ Na^+^ + OH^−^ + HOA (1)

[Nd(H_2_O)_n_]^3+^ + H_2_O ↔ [Nd(OH)(H_2_O)_n−1_]^2+^ + H_3_O^+^ (2)

[Nd(OH)(H_2_O)_n−1_]^2+^ + 2OA^−^ → Nd(OA^−^)_2_(OH)(H_2_O) + (n−3)H_2_O, (3)

where HOA = C_5_N_2_O_4_H_4_ and OA^−^ = C_5_N_2_O_4_H_3_^−^.

The complex was prepared by adding an aqueous solution of neodymium(III) salt to an aqueous solution of the sodium salt of orotic acid. The reaction mixture was stirred with an electromagnetic stirrer at 25 °C for 1 h. At the moment of mixing of the solutions, a precipitate was obtained and then filtered (the pH of the filtrate was 5.0), washed several times with water, and dried in a desiccator to a constant weight.

The complex was insoluble in water, methanol, and ethanol, and well soluble in DMSO.

### 3.2. Methods

The carbon, hydrogen, and nitrogen contents of the compound were determined via elemental analysis. The water content was determined using a Metrohm Herizall E55 Karl Fischer titrator, and via thermogravimetric analysis.

The solid-state infrared spectra of the ligand and its Nd(III) complex were recorded in KBr in the 4000–400 cm^−1^ frequency range using an FT-IR 113V Bruker spectrometer.

The Raman spectra of orotic acid and its Nd(III) complex were recorded using a Raman spectrometer LabRAM HR-800 (Horiba Jobin Yvon Gmbh, Bensheim, Germany using the 784.8 nm excitation line from a near-infrared diode laser. The LabRAM integrated system was coupled through an Olympus LMPlanFL 50× objective to the optical microscope. The spectra were collected through the backscattering geometry with a resolution of 2 cm^−1^. The detection of the Raman signals was carried out using a Peltier-cooled CCD camera. A laser power of 35 mW was used in our measurements.

### 3.3. Computational Details

The optimization and single-point calculations of the HOA, the OA anion, and the doublet state of the OA–Nd complex were performed using the Gaussian 09, revision A.02 software package [54], by using DFT approaches. For quantum chemical calculations we used the hybrid B3LYP exchange– correlation functional [55,56,57,58]. The cc-pVDZ basis set [59] was used for H, C, N, O, and Na atoms, while for the Nd atom we employed the SDD effective core potential and basis set. [60] 

This level of theory has been selected based on the fact that the hybrid B3LYP-xc functional is recognized to provide good results for a variety of molecular properties, including geometries and vibrational spectra [61]. Given the relatively large size of the complex we had to find a compromise between the available computational resources and the accuracy of the computational data. For this reason we used the valence double-zeta basis set, coupled with the SDD effective core potential recommended for lanthanides [62].

Default criteria were used to define the convergence of both the electronic density and molecular geometries. Optimized molecular geometries were used to calculate the vibrational normal modes, within the harmonic approximation. Only real wavenumbers were obtained for all structures, confirming that they correspond to true minima on the potential energy surfaces.

### 3.4. Tumour Cell Lines

Jurkat (human T-cell acute lymphoblastic leukemia, bcl-2 overexpressed), HeLa (human cervical adenocarcinoma), MCF-7 (human breast adenocarcinoma, oestrogen receptor-positive), MDA-MB-231 (human breast adenocarcinoma, oestrogen receptor-negative), and A-549 (human lung adenocarcinoma) cell lines were kindly provided by Dr. M. Hajdúch (Olomouc, Czech Republic). The CCRF-CEM cell line (human T-cell acute lymphoblastic leukemia) was obtained from the German Collection of Microorganisms and Cell Cultures (Braunschweig, Germany).

The cells were routinely maintained in a RPMI 1640 medium with L-Glutamine and HEPES (Jurkat, HeLa and CCR-CEM), or in Dulbecco’s modified Eagle’s medium with Glutamax-I (MCF-7, MDA-MB-231, and A-549), supplemented with 10% foetal calf serum, penicillin (100 IU × mL^−1^), and streptomycin (100 μg × mL^−1^) (all from Invitrogen, Waltham, MA, USA), in humidified air with 5% CO_2_ at 37 °C. Before each cytotoxicity assay, cell viability was determined using the trypan blue exclusion method, and found to be greater than 95%.

### 3.5. Cytotoxicity Assay

The cytotoxic effects of orotic acid, sodium salt of orotic acid, and the Nd(III) complex were determined using a colorimetric microculture assay with the MTT end-point [63]. Briefly, 3 × 10^3^ (A-549, MCF-7, MDA-MB-231), 5 × 10^3^ (HeLa), or 1 × 10^4^ (Jurkat and CEM) cells were plated per well in 96-well polystyrene microplates (Sarstedt, Germany) in the culture medium containing the tested chemicals at final concentrations of 10^−4^–10^−9^ mol·L^−1^. After 72 h of incubation, 10 μL of MTT (5 mg × mL^−1^) (Sigma, Darmstadt, Germany) were added to each well. After an additional 4 h, during which insoluble formazan was produced, 100 μL of 10% sodium dodecyl sulphate were added to each well, and another 12 h were allowed for the dissolution of the formazan. Absorbance was measured at 540 nm using an automated MRX microplate reader (Dynatech Laboratories, Chichester, UK). The blank-corrected absorbance of the control wells was taken as 100%, and the results were expressed as a percentage of the control. All experiments were performed in triplicate.

## 4. Conclusions

The complex of neodymium(III) with orotic acid has been synthesized and characterized via elemental and vibrational (IR, Raman) analyses. The vibrational analysis performed for the studied species helped to explain the vibrational behaviour of the ligand vibrational modes, sensitive to interaction with Nd(III). The most probable metal–ligand binding mode in the Nd(III) complex of orotic acid was elucidated. It is suggested that orotic acid binds through the oxygen atoms of the carboxylic groups from both the ligands, and from one OH^−^ ion. One of the two OA anions is monodentate, while the second one is in bidentate form, bound to the Nd(III) ion through the carboxylic oxygens.

The results from the preliminary cytotoxic screening of orotic acid, sodium salt of orotic acid, and the Nd(III) complex demonstrate the anti-proliferative potential of the Nd(III) complex, which is in line with our preceding papers concerning the activity of lanthanide coordination compounds with diverse biologically active ligands. The complex formation proved to be beneficial for the efficacy of the Nd(III) complex, as the complex exerted efficacy vs. the corresponding ligands on all of the cell lines. Thus, this matter necessitates further, more detailed pharmacological evaluation. Additionally, the toxicology of the investigated compounds is of great interest with respect to their further pharmacological properties, and will be the subject of coming investigations.

## Figures and Tables

**Table 1:** Calculated Nd–O distances and APT partial atomic charges of the Nd(III) complex of orotic acid at the B3LYP/cc-pVDZ(SDD) level of theory.

Nd–O Distances	Calculated Value (Å)
Nd–O1	2.155
Nd–O1′	2.381
Nd–O3′	2.441
Nd–O5	2.061
APT partial atomic charges	Calculated value (e)
Nd	+2.812
O1	−1.558
O1′	−1.109
O3′	−1.101
O5	−1.186

**Table 2:** Selected theoretical B3LYP-cc-pVDZ(SDD) and experimental IR and Raman wavenumbers (cm^−1^) of the Nd(III) complex (NdOA), and their tentative assignment. Experimental data for orotic acid (HOA) and its anion (OA^−^) are included for comparative purposes.

Mode nr.	Scaled Wave Numbers (cm^−1^)	Exp. Raman			Exp. IR			Assignments
		HOA	OA-	NdOA	HOA	OA-	NdOA	
1	190	-	-	-	-	-	-	δ(ONdO)
2	265	-	-	-	-	-	-	ν(O1–Nd), δ(C6–C7–O1)
3	278	-	-	-	-	-	-	ν(O3′–Nd), δ(C6′–C7′–O3′)
4	459	-	-	-	-	435	442	ν(O1′–Nd), ν(O3′–Nd), δ(C6′–C7′–O1′), δ(C6′–C7′–O3′)
5	466	460	459	461	451	-	-	δ(C6–C7–O1), δ(C7–O1–Nd), δ(N1–C2–O2)
6	514	513	514	510	507	509	517	δ(O5–H6), γ(N1–C6)
7	535	546	551	539	555	555	548	δ(C6–C5–C4), δ(C5–C4–N3), δ(N3–C4–O4), δ(C2–N1–C6)
8	610	608	605	595	603	600	595	ν(Nd–O5)
9	657	655	642	645	-	639	643	γ(N1–H1), γ(N3–H3), γ(C5–H5)
10	765	753	-	-	758	758	757	γ(C2′O), γ(C2′N)
11	788	-	780	779	774	779	771	δ(C7O_2_), ν(NdO1)
12	798	793	-	794	785	796	799	δ(C7′O_2_)
13	886	887	856	852	892	853	859	γ(C4′–H5′), γ(C4′–C5′), γ(C5′–C6′), γ(C6′–N1′)
14	931	932	934	933	930	929	933	ν(C6′–C7′), δ(C7′O_2_), δ(C2′–N3′–C4′)
15	1012	1011	1018	1006	1015	1018	1015	δ(C2′–N1′–C6′), δ(N1′–C6′–C5′), δ(C4′–C5′–C6′)
16	1032	1047	1048	1032	1042	1042	1036	δ(C5′–C6′–N1′), δ(C6′–N1′–C2′), ν(C6′–C7′)
17	1085	1135	1117	-	1125	-	1121	β(C5–H5), δ(C5C6N1), ν(Nd–O1′)
18	1215	1255	1239	1226	1241	1234	1232	ν(C7–O1)
19	1292	1282	1294	1295	1284	-	-	β(N1–H1), β(C5–H5), ν(C6–C7)
20	1347	1326	-	-	1345	1322	1311	δ(N3–H3)
21	1384	-	1386	1389	-	1386	1384	δ(N3C2O2), ν(C2N1), δ(C2N1H1)
22	1399	1414	1428	1410	1407	1426	1407	ν(C6′–C7′), ν_s_(C7′O_2_)
23	1521	1522	1498	-	1525	1497	-	ν_as_(C7′O_2_)
24	1626	1615	-	1615	1617	1620	1602	ν(C5–C6)
25	1638	1657	1652	1667	1655	1654	1653	ν(C6′–C5′)
26	1745	-	1684	1688	1684	1696	1684	ν(C7O3), ν(C4O4)
27	1753	1713	1710	1714	1712	1707	1728	ν(C2O2)
28	1783	-	1746	1744	1734	1741	1743	ν(C2′O2′)
29	3164	-	-	3108	-	3116	-	ν(C5–H5)
30	3386	-	-	-	3520	3214	3417	ν(N1–H1)
31	3682	3144	-	-	3232	-	3201	ν(O5–H6)

**Table 3:** Cytotoxicity of the studied compounds at a concentration of 10^−4^ mol·L^−1^ (percentage of living cells compared to solvent control (100%)).

Compound	Cancer Cell Lines					
	Jurkat	CCRF-CEM	HeLa	A-549	MCF	MDA
HOA	63.3	61.2	84.7	96.9	93.9	93.76
NaOA	58.6	64.8	78.6	90.6	100.0	93.7
NdOA	67.3	59.8	69.4	91.8	100	83.1

Abbreviation: Jurkat cells (acute T-lymphoblastic leukemia, bcl-2 overexpressed); CCRF-CEM (acute T-lymphoblastic leukemia); HeLa (human cervical adenocarcinoma); A-549 (lung carcinoma); MCF-7 (mammary gland adenocarcinoma, oestrogen receptor expressed); and MDA-MB-231 (human breast adenocarcinoma, oestrogen receptor-negative).

## Sample Availability

A sample of the synthesized complex is available from the authors upon request.
